# TGF-β1 and TGFβR2 Gene Polymorphisms in Patients with Unstable Angina

**DOI:** 10.3390/biomedicines11010155

**Published:** 2023-01-07

**Authors:** Damian Malinowski, Krzysztof Safranow, Andrzej Pawlik

**Affiliations:** 1 Department of Pharmacokinetics and Therapeutic Drug Monitoring, Pomeranian Medical University, 70-111 Szczecin, Poland; 2 Department of Biochemistry and Medical Chemistry, Pomeranian Medical University, 70-111 Szczecin, Poland; 3 Department of Physiology, Pomeranian Medical University, 70-111 Szczecin, Poland

**Keywords:** coronary artery disease, unstable angina, polymorphism, genetic, transforming growth factor

## Abstract

Acute coronary syndromes result from a sudden reduction in the lumen of a coronary artery as a result of atherosclerotic plaque rupture, its swelling or the formation of thrombotic lesions. Many mediators with inflammatory, prothrombotic and proatherogenic effects have been shown to be involved, including numerous cytokines, chemokines, adhesion molecules and growth factors. TGF-β1 is a pleiotropic cytokine found in various cells that regulates cell growth, differentiation and matrix production. The aim of our study was to assess the association between polymorphisms in the *TGF-β1* gene (rs1800469, rs1800470) and polymorphisms in the *TGFBR2* receptor gene (rs6785358, rs9838682) and the risk of unstable angina, as well as selected clinical parameters affecting the risk of ischemic heart disease. The study included 232 patients with unstable angina. The diagnosis of unstable angina was made by typical clinical presentation and confirmation of significant coronary artery lumen stenosis (>70%) during coronary angiography. There were no statistically significant differences in the distribution of *TGFBR2* rs6785358 and rs9838682 genotypes and haplotypes between patients with unstable angina and control subjects. We observed increased values of plasma total and LDL cholesterol levels, as well as triglycerides, in patients with the *TGFBR2* rs9838682 AA genotype. In patients with the *TGFBR2* rs6785358 AA genotype, we noted increased BMI values. There were no statistically significant associations between other studied polymorphisms and clinical parameters. Polymorphisms in the *TGF-β1* gene (rs1800469, rs1800470) and polymorphisms in the *TGFBR2* receptor gene (rs6785358, rs9838682) are not significant risk factors for unstable angina in our population. The *TGFBR2* gene rs9838682 polymorphism may influence the lipid parameters in patients with coronary artery disease.

## 1. Introduction

Acute coronary syndromes result from a sudden decrease in the lumen of the coronary artery due to atherosclerotic plaque rupture, its swelling or the formation of thrombotic lesions. A number of mediators have been shown to be involved which have post-inflammatory, procoagulant and proatherogenic effects, including numerous cytokines, chemokines, adhesion molecules and growth factors. TGF-β1 is a pleiotropic cytokine found in various cells, including haematopoietic, connective tissue and endothelial cells. It regulates cell growth, differentiation and matrix production [1,2,3].

The TGF-β receptor (TGFBR), including TGFBR1, TGFBR2 and TGFBR3, is a serine/threonine protein kinase present on the cell surface. Activation of TGF-β1-related signalling pathways occurs by the binding of TGF-β1 to its receptor [1,2,3].

There is increasing evidence that TGF-β1 is involved in the development of atherosclerosis and ischemic heart disease. However, it has not been fully clarified whether TGF-β1 has pro- or antiatherogenic effects. TGF-β1 is generally considered to be an anti-inflammatory cytokine. However, it has been shown that TGF-β1 can enhance the atherogenic process by increasing the expression of pro-atherogenic genes [3,4,5,6]. TGF-β1 is a cytokine with pleiotropic effects. In the cardiovascular system, TGF-β induces neoangiogenesis, cardiomyocyte hypertrophy and fibrosis [7]. Although environmental factors play an important role in the development of atherosclerosis, a genetic influence on this process is increasingly being considered. The involvement of TGF-β1 has been confirmed in the pathogenesis of many diseases, such as cancer and vascular disease [8]. It has been shown that certain polymorphisms present within the TGF-β1 gene can affect its expression and TGF-β1 protein synthesis. One of the most studied genetic variants of *TGF-β1* is the T29C polymorphism (rs1800470), resulting from the substitution of proline (CCG) for leucine (CTG) at codon 10 (Pro10Leu) of the protein [9,10]. Other polymorphisms of the promoter region of the *TGFB1* gene affecting its expression include the rs1800469 polymorphism (−509 C/T). This polymorphism has been shown to affect the amount of TGF-β1 protein produced. Carriers of the C allele are characterized by transcriptional suppression through AP1 (Activator protein 1) binding, resulting in reduced TGF-β1 protein synthesis [11]. Some studies suggest that these polymorphisms are associated with coronary atherosclerosis [12,13]. It has also been shown that the presence of rs9838682 and rs6785358 polymorphisms in the TGF-β1 receptor region may affect TGF-β1-related signalling pathways and may be associated with an increased risk of coronary heart disease [14]. The TGF-β1 receptor gene rs9838682 polymorphism is located on chromosome 3 in position 30365871. This SNP is intronic and is located between exons 3 and 4. It can probably regulate gene splicing and transcription.

The aim of our study was to assess the association between polymorphisms in the *TGF-β1* gene (rs1800469, rs1800470) and in the *TGFBR2* gene (rs6785358, rs9838682) and the risk of unstable angina, as well as selected clinical parameters affecting the risk of ischemic heart disease.

## 2. Materials and Methods

The study included 232 individuals with unstable angina (age 62.07 ± 9.68 years) (Table 1). The diagnosis of unstable angina was made by typical clinical presentation (angina at rest associated with acute or transient ST segment or T wave changes in ECG without an increase in markers of myocardial injury, i.e., troponin T, myoglobin) and confirmation of significant coronary artery lumen stenosis (>70%) during coronary angiography. The criteria for patient exclusion from the study were a final diagnosis of myocardial infarction based on a significant increase in markers of myocardial injury (troponin T, myoglobin), autoimmune diseases and cancer.

The control group consisted of 144 healthy subjects (age 67.4 ± 10.6 years) without a history of inflammatory disease or cancer (Table 1). In this group of patients, no significant coronary lumen stenosis was detected during coronary angiography (performed for a diagnosis of unexplained pain in chest). The study was approved by the local ethics committee at Pomeranian Medical University, Szczecin, Poland (KB-0012/46/17). Written informed consent was obtained from all participants.

### 2.1. Genotyping

Genomic DNA was extracted from 1 mL of peripheral blood samples (collected in EDTA tubes) using a Genomic Mini AX Blood 1000 Spin kit (A&A Biotechnology, Gdynia, Poland) according to the manufacturer’s protocol. Prior to the isolation, blood samples were stored at −80 °C. Extracted DNA was subsequently standardized to equal concentrations of 20 ng/µL based on spectrophotometric absorbance measurement (260/280 nm) using a DeNovix DS-11 FX+ Spectrophotometer/Fluorometer (Wilmington, DE, USA).

Genotyping for the following single nucleotide polymorphisms (SNPs): *TGFB1* rs1800469, rs1800470, *TGFBR2* rs6785358 and rs9838682 was performed using pre-validated allelic discrimination TaqMan real-time PCR assays (Life Technologies, Waltham, MA, USA; TaqMan Assay ID’s: C___8708473_10, C__22272997_10, C___1981957_10, C__11907338_10) and TaqMan GTXpress Master Mix (Life Technologies, Waltham, MA, USA) (Table 2). All reactions were run in duplicate in a final volume of 12 µL (reaction temperature profile: 95 °C, 20 s; 40 × 95 °C 1 s/60 °C, 20 s; 40-cycle reaction).

Genotyping was performed using the ViiA7 Real-Time PCR System (Applied Biosystems, Waltham, MA, USA). Genotypes were assigned to individual samples after the analysis with TaqMan Genotyper software (Thermo Fisher Scientific, Waltham, MA, USA).

### 2.2. Statistical Analysis

The concordance of genotype distributions with Hardy-Weinberg equilibrium (HWE) was assessed using Fisher’s exact test. The χ^2^ test was used to compare the distributions of genotypes and alleles between groups. The distribution of quantitative clinical parameters in the study group differed significantly from the normal distribution (Shapiro-Wilk test), thus they were compared between groups using the non-parametric Mann-Whitney test. A *p*-value < 0.05 was considered statistically significant. Statistical analysis was performed using STATISTICA PL, ver. 13.1 software (StatSoft, Inc., Tulsa, OK, USA, 2016, STATISTICA data analysis software system). Haplotype reconstruction and linkage disequilibrium analysis were performed by means of HaploView 4.2 software (Broad Institute, Cambridge, MA, USA).

## 3. Results

The distribution of the studied polymorphisms in patients with unstable angina and the control group are in HWE and are shown in Table 3. As seen in Table 3, there were no statistically significant differences in the distribution of the studied polymorphisms between patients with unstable angina and control subjects.

Additionally, we compared the distribution of the studied polymorphisms in groups of patients younger and older than 55 years (Supplementary Tables S1 and S2). In both these groups, there were no statistically significant differences in the distribution of the studied polymorphisms between patients with unstable angina and healthy controls.

We also compared the haplotype frequency of *TGFBR2* rs6785358 and rs9838682 in unstable angina and the control group (Table 4). These differences were statistically non-significant.

The next step of our study was to examine the associations between the studied polymorphisms and selected clinical parameters in patients with unstable angina (Table 5, Table 6, Table 7 and Table 8). We observed increased values of plasma total and LDL cholesterol levels, as well as triglycerides, in patients with the *TGFBR2* rs9838682 AA genotype. In patients with the *TGFBR2* rs6785358 AA genotype, we noted increased BMI values. There were no statistically significant associations between the other studied polymorphisms and clinical parameters.

We also examined the distribution of the studied polymorphisms between patients with and without diabetes mellitus as well as with and without hypertension. These differences were statistically non-significant (Supplementary Tables S3 and S4).

## 4. Discussion

The aim of this study was to examine the association between polymorphisms in the *TGF-β1* gene (rs1800469, rs1800470) and polymorphisms in the *TGFBR2* receptor gene (rs6785358, rs9838682) and the risk of unstable angina, as well as selected clinical parameters affecting the risk of ischemic heart disease. We found no differences in the distribution of the polymorphisms studied between patients with unstable angina and controls, suggesting that these polymorphisms are not factors associated with the risk of developing unstable angina in our population. We found increased lipid metabolism parameters (total LDL cholesterol, triglycerides) in patients with *TGFBR2* rs9838682 AA genotype.

The TGF-β signalling pathway plays a key role in the regulation of many cellular processes, such as cell proliferation and differentiation, extracellular matrix production, cell adhesion and apoptosis [7,8]. Abnormalities in the TGF-β signalling pathway have been found to lead to a variety of human diseases, such as hypertension, hyperlipidaemia, atherosclerosis and renal fibrosis [7,8,15]. TGF-β exerts its action through TGF-β type I and TGF-β type II receptors. TGF-β binds first bind to type II receptors, which then form a ligand-receptor complex with type I receptors. The TGF-β type I receptor directly activates the intracellular proteins SMAD2 and SMAD3 through their phosphorylation, which then mediate TGF-β signalling [8].

Cholesterol is one of the main causative factors in the development of atherosclerotic lesions and cardiovascular disease. The mechanisms by which cholesterol initiates atherogenesis have been extensively studied but remain poorly understood. Hypercholesterolemia has been shown to inhibit TGF-β expression in endothelial cells, leading to the development of atherosclerotic lesions [16]. The findings indicate that TGF-β1 may have an anti-inflammatory role in the vessel wall. TGF-β1 in macrophages has been shown to cause a reduction and stabilisation of atherosclerotic plaque in ApoE-deficient mice [17]. Activation of TGF-β1 signalling pathways led to the overexpression of the pluripotent proteoglycan V3 in arterial smooth muscle cells, thereby reducing platelet adhesion to blood vessels [18].

Previous studies have shown that inflammation plays an important role in the development of the atherosclerotic process [19]. In the early stages of atherosclerosis there is damage of the endothelium and activation of inflammatory mediators, which include monocyte chemoattractant protein-1, IL-8 and adhesion molecules ICAM-1, VCAM-1 and E-selectin. Many cells such as macrophages, neutrophils, T and B lymphocytes, dendritic cells, endothelial cells and vascular smooth muscle cells are involved in this process [19]. A large number of low-density lipoproteins (LDL) are modified to oxidized LDL (oxLDL) and accumulate in the inner vessel wall, contributing to the development of the atherosclerotic plaque. Monocytes differentiate into macrophages, which absorb oxLDL and transform into foam cells [20]. Numerous mediators secreted by immune cells and vascular endothelial cells activate and sustain inflammation in the vessels, exacerbating the development of atherosclerotic lesions. The atherogenic process begins with the accumulation of plasma lipoproteins in the subendothelial space. In the intima, LDL undergoes oxidative modification by reactive oxygen species (ROS), which promotes the uptake of oxLDL by macrophages. In addition, oxidized phospholipids induce inflammation in the arterial wall by binding to Toll-like receptors, which enhance the inflammatory process [21]. The cells that play an important role in atherogenesis are neutrophils. They are the source of metalloproteinases MMP-8 and -9, myeloperoxidase and neutrophil elastase, which can cause plaque instability [22]. Recently, neutrophil extracellular traps (NETs) have been recognized as an additional defence mechanism in a process called NETosis [23,24]. Neutrophil extracellular traps are formed by chromatin, histones and granular proteins of neutrophils, and play a role in trapping microbial pathogens. However, NETs also have prothrombotic properties by stimulating fibrin deposition, and increased levels of NETs correlate with higher rates of cardiovascular complications [25,26].

Previous studies have examined the associations between *TGF-β* gene polymorphisms and coronary artery disease; however, the results are conflicting and differ between populations. The *TGFB1* gene rs1800469 polymorphism was associated with myocardial infarction in men, independently from the potentially confounding factors: age, arterial hypertension, hypercholesterolemia, cigarette smoking and diabetes mellitus. Lower risks of myocardial infarction were observed among the carriers of the CC genotype in a German population [27]. The results of studies from the United Kingdom have shown that *TGFB1* rs1800470 is associated with ischemic heart disease in patients with rheumatoid arthritis [28]. Also, in a Chinese population, *TGFB1* rs1800469 and *TGFB1* rs1800470 were associated with coronary artery disease [29]. Yang et al. indicated that the *TGF-β1* rs1800470 gene polymorphism may be associated with the severity of coronary artery disease in male patients [30]. In contrast, a study carried out in a Spanish population did not find statistically significant associations between *TGFB1* rs1800469 and coronary artery disease [31]. A meta-analysis has shown that the pooled odds ratios for coronary heart disease among minor T allele carriers of rs1800469 versus homozygous major allele carriers were significantly increased [32]. It has been shown that angiographically significant in-stent restenosis was significantly less frequently observed in the group of patients with the AA genotype of rs1800470 polymorphism (*TGFB1*) versus patients with AG and GG genotypes [33].

Morris et al. performed a meta-analysis of published case-control studies assessing the association of *TGF-β* SNPs with a range of coronary heart disease complications [14]. A random effects model was used to calculate odds ratios and confidence intervals. Analyses were conducted for additive, dominant and recessive modes of inheritance. Applying a dominant model of inheritance, the *TGF-β1* T alleles of rs1800469 and rs1800470 were significantly associated with coronary heart disease complications. Fragoso et al. examined the role of *TGF-β1* gene polymorphisms in the risk of developing in-stent restenosis [34]. The *TGF-β1* rs1800469 and rs1800470 gene polymorphisms were analysed. The *TGF-β1* rs1800470 polymorphism was significantly associated with an increased risk of restenosis. It has been also shown that lower serum TGF-β1 levels are associated with an increased risk for coronary artery ectasia development. Furthermore, *TGF-β1* rs1800470 G allele carriers were shown to have higher TGF-β1 levels in the coronary artery ectasia group.

*TGFβR2* gene polymorphisms have not been widely examined in patients with diseases of the circulatory system. Huang et al. suggested that the *TGFBR2* gene rs6785358 polymorphism is associated with an increased risk of congenital heart defects in Han Chinese men [35]. The *TGFBR2* gene rs6785358 polymorphism was also associated with an increased risk of congenital ventricular septal defect in G allele carriers compared with A allele carriers. Tseng et al. found that the *TGFBR2* rs9838682 polymorphism was associated with the risk of sudden cardiac arrest in patients with coronary artery disease [36]. Our results showed increased levels of total cholesterol, LDL and triglycerides in patients with rs9838682 AA genotype. Previous studies have indicated that TGFBR2 signalling is involved in regulating lipid metabolism [37,38]. In addition, the rs9838682 polymorphism of the *TGFBR2* gene may be in linkage disequilibrium with other functional variants affecting lipid metabolism. However, limited data are available on its specific functional effects, so further studies are needed to understand the role of this polymorphism in regulating metabolic processes. Our study has some limitations, such as the lack of TGF assays in serum, but we hope that it will prompt further research into the role of *TGF-β1* and its receptors in the pathogenesis of coronary artery disease.

The results of our study suggest that polymorphisms in the *TGF-β1* gene (rs1800469, rs1800470) and polymorphisms in the *TGFBR2* receptor gene (rs6785358, rs9838682) are not the significant risk factors of unstable angina in our population. However, the *TGFBR2* gene rs9838682 polymorphism may affect lipid metabolism parameters.

Carriers of the *TGFBR2* rs9838682 AA genotype may have increased serum total cholesterol, LDL cholesterol and triglyceride levels.

## 5. Conclusions

Polymorphisms in the *TGF-β1* gene (rs1800469, rs1800470) and in the *TGFBR2* receptor gene (rs6785358, rs9838682) are not significant risk factors for unstable angina in our population. The *TGFBR2* gene rs9838682 polymorphism may influence lipid parameters in patients with coronary artery disease.

## Figures and Tables

**Table 1:** Clinical characteristics of patients and control subjects.

Parameters	Control Group	Unstable Angina	*p* ^#^
	Mean ± SD	Mean ± SD	
Age [years]	67.44 ± 10.62	62.07 ± 9.68	<0.00001
BMI [kg/m^2^]	25.96 ± 3.64	28.37 ± 3.95	<0.00001
CH [mg/dL]	197.46 ± 40.98	230.27 ± 56.21	<0.00001
HDL [mg/dL]	53.04 ± 6.77	44.77 ± 8.40	<0.00001
LDL [mg/dL]	118.18 ± 36.84	163.70 ± 50.50	<0.00001
TG [mg/dL]	105.09 ± 45.92	139.77 ± 73.29	<0.00001

^#^ Mann-Whitney U test.

**Table 2:** TaqMan^®^ assays.

SNP	Gene Name	Gene Symbol	SNP Location	Nucleotide Change	TaqMan Assay ID
rs1800469	transforming growth factor beta 1	*TGFB1*	19q13.2	A>G	C___8708473_10
rs1800470	transforming growth factor beta 1	*TGFB1*	19q13.2	G>A	C__22272997_10
rs6785358	transforming growth factor beta receptor 2	*TGFBR2*	3p24.1	A>G	C___1981957_10
rs9838682	transforming growth factor beta receptor 2	*TGFBR2*	3p24.1	A>G	C__11907338_10

**Table 3:** Distribution of *TGFB1* rs1800469, rs1800470, and *TGFBR2* rs6785358, rs9838682 genotypes and alleles in patients with unstable angina and controls.

	Control Group (*n* = 144)		Unstable Angina (*n* = 232)		*p*-Value ^^^	Compared Genotypes or Alleles	*p*-Value ^#^	OR (95% CI)
	*n*	%	*n*	%				
***TGFB1 rs1800469***								
**genotype**								
GG	62	43.06%	86	37.07%	0.454	AA+GA vs. GG	0.28	1.28 (0.84–1.96)
GA	64	44.44%	118	50.86%		AA vs. GA+GG	1.00	0.96 (0.51–1.81)
AA	18	12.50%	28	12.07%		AA vs. GG	0.86	1.12 (0.57–2.21)
						GA vs. GG	0.26	1.33 (0.85–2.08)
						AA vs. GA	0.61	0.84 (0.43–1.64)
**Allele**								
G	188	65.28%	290	62.50%		A vs. G	0.48	1.13 (0.83–1.53)
A	100	34.72%	174	37.50%				
**Multivariate logistic analysis (allele)**								
*TGFB1 rs1800469*							0.69	1.07 (0.76–0.1.51)
age						A vs. G	0.0002	0.96 (0.94–0.98)
sex							<0.0001	4.26 (2.69–6.73)
***TGFB1 rs1800470*** **genotype**								
GG	24	16.67%	48	20.69%	0.621	GG+GA vs. AA	0.65	1.11 (0.72–1.73)
GA	70	48.61%	109	46.98%		GG vs. GA+AA	0.35	1.30 (0.76–2.24)
AA	50	34.72%	75	32.33%		GG vs. AA	0.37	1.33 (0.73–2.45)
						GA vs. AA	0.91	1.04 (0.65–1.66)
						GG vs. GA	0.47	1.28 (0.72–2.28)
**Allele**								
G	118	40.97%	205	44.18%		G vs. A	0.41	1.14 (0.85–1.54)
A	170	59.03%	259	55.82%				
**Multivariate logistic analysis (allele)**								
*TGFB1 rs1800470*							0.53	1.11 (0.81–1.53)
age						G vs. A	0.0002	0.96 (0.94–0.98)
sex							<0.0001	4.24 (2.68–6.71)
***TGFBR2* rs6785358 genotype**								
AA	97	67.36%	173	74.57%	0.161	GG+AG vs. AA	0.16	0.70 (0.45–1.11)
AG	45	31.25%	53	22.84%		GG vs. AG+AA	0.72	1.89 (0.38–9.47)
GG	2	1.39%	6	2.59%		GG vs. AA	0.72	1.68 (0.33–8.50)
						AG vs. AA	0.09	0.66 (0.41–1.06)
						GG vs. AG	0.30	2.55 (0.49–13.25)
**Allele**								
A	239	82.99%	399	85.99%		G vs. A	0.30	0.80 (0.53–1.19)
G	49	17.01%	65	14.01%				
**Multivariate logistic analysis (allele)**								
*TGFBR2* rs6785358							0.24	0.77 (0.49–1.20)
age						G vs. A	0.0002	0.96 (0.94–0.98)
sex							<0.0001	4.29 (2.71–6.81)
***TGFBR2* rs9838682 genotype**								
AA	20	13.89%	24	10.34%		AA+AG vs. GG	0.39	0.81 (0.53–1.24)
AG	68	47.22%	106	45.69%	0.462	AA vs. AG+GG	0.32	0.72 (0.38–1.35)
GG	56	38.89%	102	43.97%		AA vs. GG	0.29	0.66 (0.34–1.30)
						AG vs. GG	0.50	0.86 (0.55–1.34)
**Allele**						AA vs. AG	0.49	0.77 (0.40–1.50)
A	108	37.50%	154	33.19%				
G	180	62.50%	310	66.81%		A vs. G	0.24	0.83 (0.61–1.13)
**Multivariate logistic analysis (allele)**								
*TGFBR2* rs9838682							0.15	0.78 (0.55–1.09)
age						A vs. G	0.0001	0.96 (0.93–0.98)
sex							<0.0001	4.26 (2.69–6.75)

^ χ^2^ test; ^#^ Fisher’s Exact Test; HWE: control group *p* = 0.854, unstable angina *p* = 0.262 for *TGFB1* rs1800469; HWE: control group *p* = 1.00, unstable angina *p* = 0.506 for *TGFB1* rs1800470; HWE: control group *p* = 0.371, unstable angina *p* = 0.414 for *TGFBR2* rs6785358; HWE: control group *p* = 1.00, unstable angina *p* = 0.767 for *TGFBR2* rs9838682.

**Table 4:** Haplotype frequency of *TGFBR2* rs6785358 and rs9838682 in unstable angina and control group.

Haplotype	Control Group	Unstable Angina	*p* ^a^
GA	0.525	0.560	0.59
AA	0.305	0.300	1.00
GG	0.100	0.108	0.86
AG	0.070	0.032	0.12

^a^ Fisher’s Exact Test.

**Table 5:** Associations between the clinical parameters of patients with unstable angina and the *TGFB1* rs1800469 genotypes.

Parameters	*TGFB1* rs1800469 Genotype										
		GG		GA		AA	GG vs. GA	GA vs. AA	GG vs. AA	AA+GA vs. GG	GG+GA vs. AA
	*n*	Mean ± SD	*n*	Mean ± SD	*n*	Mean ± SD	*p* ^&^				
Age [years]	86	63.45 ± 9.20	118	61.12 ± 10.16	28	61.79 ± 8.76	0.099	0.616	0.525	0.112	0.990
BMI [kg/m^2^]	86	28.35 ± 3.91	118	28.56 ± 4.01	28	27.64 ± 3.93	0.833	0.283	0.355	0.916	0.284
CH [mg/dL]	82	225.35 ± 50.39	114	236.39 ± 60.27	27	219.41 ± 54.01	0.266	0.144	0.331	0.523	0.184
HDL [mg/dL]	70	43.97 ± 7.76	93	45.58 ± 8.93	23	43.91 ± 8.12	0.277	0.389	0.993	0.364	0.602
LDL [mg/dL]	70	157.74 ± 45.45	93	171.25 ± 53.41	23	151.35 ± 50.21	0.084	0.098	0.413	0.234	0.170
TG [mg/dL]	81	147.57 ± 98.28	114	138.28 ± 53.76	27	122.63 ± 53.59	0.511	0.131	0.381	0.776	0.188

^&^—Mann–Whitney U test; BMI—body mass index; CH—total cholesterol in serum; HDL—high density cholesterol in serum; LDL—low density cholesterol in serum; TG—triacylglycerols in serum.

**Table 6:** Associations between the clinical parameters of patients with unstable angina and the *TGFB1* rs1800470 genotypes.

Parameters	*TGFB1* rs1800470 Genotype										
		GG		GA		AA	AA vs. GA	GA vs. GG	GG vs. AA	AA+GA vs. GG	GG+GA vs. AA
	*n*	Mean ± SD	*n*	Mean ± SD	*n*	Mean ± SD	*p* ^&^				
Age [years]	48	62.54 ± 9.26	109	60.94 ± 10.18	75	63.40 ± 9.10	0.085	0.213	0.854	0.483	0.176
BMI [kg/m^2^]	48	27.75 ± 3.42	109	28.74 ± 4.14	75	28.23 ± 3.98	0.463	0.174	0.527	0.247	0.771
CH [mg/dL]	47	231.00 ± 60.45	105	231.71 ± 56.94	71	227.66 ± 52.84	0.760	0.854	0.878	0.850	0.871
HDL [mg/dL]	39	43.85 ± 8.27	87	45.71 ± 8.95	60	44.00 ± 7.62	0.308	0.272	0.903	0.450	0.482
LDL [mg/dL]	39	162.92 ± 56.07	87	165.23 ± 49.77	60	162.00 ± 48.51	0.665	0.688	0.926	0.765	0.779
TG [mg/dL]	47	124.06 ± 51.11	105	139.00 ± 55.13	70	151.46 ± 102.83	0.658	0.120	0.316	0.144	0.938

^&^—Mann–Whitney U test; BMI—body mass index; CH—total cholesterol in serum; HDL—high density cholesterol in serum; LDL—low density cholesterol in serum; TG—triacylglycerols in serum.

**Table 7:** Associations between the clinical parameters of patients with unstable angina and the *TGFBR2* rs6785358 genotypes.

Parameters	*TGFBR2* rs6785358 Genotype										
		AA		AG		GG	AA vs. AG	GG vs. AG	AA vs. GG	AA+GA vs. GG	AG+GG vs. AA
	*n*	Mean ± SD	*n*	Mean ± SD	*n*	Mean ± SD	*p* ^&^				
Age [years]	173	61.84 ± 9.52	53	62.47 ± 10.07	6	64.83 ± 11.79	0.714	0.670	0.583	0.596	0.619
BMI [kg/m^2^]	173	28.68 ± 3.98	53	27.55 ± 3.82	6	26.83 ± 3.31	0.075	0.604	0.247	0.307	0.046
CH [mg/dL]	165	231.67 ± 54.15	53	227.77 ± 63.22	5	210.80 ± 50.38	0.398	0.406	0.393	0.388	0.308
HDL [mg/dL]	141	44.79 ± 8.62	40	44.83 ± 7.62	5	43.60 ± 9.94	0.932	0.786	0.813	0.800	0.994
LDL [mg/dL]	141	163.77 ± 48.10	40	166.05 ± 59.34	5	143.60 ± 45.31	0.959	0.437	0.454	0.441	0.864
TG [mg/dL]	164	138.71 ± 67.10	53	147.00 ± 91.90	5	97.80 ± 24.03	0.844	0.124	0.136	0.127	0.845

^&^—Mann–Whitney U test; BMI—body mass index; CH—total cholesterol in serum; HDL—high density cholesterol in serum; LDL—low density cholesterol in serum; TG—triacylglycerols in serum.

**Table 8:** Associations between the clinical parameters of patients with unstable angina and the *TGFBR2 rs9838682* genotypes.

Parameters	*TGFBR2* rs9838682 Genotype										
		AA		AG		GG	GG vs. AG	AG vs. AA	GG vs. AA	GG+AG vs. AA	AA+AG vs. GG
	*n*	Mean ± SD	*n*	Mean ± SD	*n*	Mean ± SD			*p* ^&^		
Age [years]	24	63.17 ± 10.47	106	61.30 ± 9.67	102	62.60 ± 9.54	0.296	0.423	0.821	0.584	0.412
BMI [kg/m^2^]	24	29.33 ± 3.98	106	28.60 ± 3.87	102	27.90 ± 4.01	0.180	0.322	0.068	0.140	0.084
CH [mg/dL]	24	246.63 ± 43.85	102	227.74 ± 58.94	97	228.90 ± 55.81	0.709	0.047	0.048	0.036	0.750
HDL [mg/dL]	21	42.38 ± 8.98	78	44.96 ± 9.34	87	45.17 ± 7.32	0.552	0.239	0.091	0.126	0.274
LDL [mg/dL]	21	182.19 ± 42.84	78	164.09 ± 52.74	87	158.90 ± 49.61	0.592	0.081	0.037	0.042	0.237
TG [mg/dL]	24	159.50 ± 66.75	101	140.58 ± 74.59	97	134.03 ± 73.29	0.440	0.091	0.033	0.044	0.178

^&^—Mann–Whitney U test; BMI—body mass index; CH—total cholesterol in serum; HDL—high density cholesterol in serum; LDL—low density cholesterol in serum; TG—triacylglycerols in serum.

## Data Availability

Not applicable.

## Supplementary Materials

The following supporting information can be downloaded at: https://www.mdpi.com/article/10.3390/biomedicines11010155/s1, Table S1. Distribution of *TGFB1* rs1800469, rs1800470, *TGFBR2* rs6785358, rs9838682 genotypes and alleles in with unstable angina and controls in <55 years group; Table S2. Distribution of *TGFB1* rs1800469, rs1800470, *TGFBR2* rs6785358, rs9838682 genotypes and alleles in with unstable angina and controls in ≥55 years group; Table S3. Distributions of the *TGFB1* rs1800469, rs1800470, *TGFBR2* rs6785358, rs9838682 genotypes and alleles in unstable angina patients with and without diabetes mellitus (DM); Table S4. Distributions of the *TGFB1* rs1800469, rs1800470, *TGFBR2* rs6785358, rs9838682 genotypes and alleles in unstable angina patients with and without arterial hypertension (HA).

## References

[1] Goumans M.J., Ten Dijke P. (2018). TGF-β Signaling in Control of Cardiovascular Function. Cold Spring Harb. Perspect. Biol..

[2] Dobaczewski M., Chen W., Frangogiannis N.G. (2011). Transforming growth factor (TGF)-β signaling in cardiac remodeling. J. Mol. Cell. Cardiol..

[3] Sorensen D.W., van Berlo J.H. (2020). The Role of TGF-β Signaling in Cardiomyocyte Proliferation. Curr. Heart Fail. Rep..

[4] Yu X.H., Fu Y.C., Zhang D.W., Yin K., Tang C.K. (2013). Foam cells in atherosclerosis. Clin. Chim. Acta..

[5] Tedgui A., Mallat Z. (2006). Cytokines in atherosclerosis: Pathogenic and regulatory pathways. Physiol. Rev..

[6] Grainger D.J. (2007). TGF-beta and atherosclerosis in man. Cardiovasc. Res..

[7] Blann A.D., Wang J.M., Wilson P.B., Kumar S. (1996). Serum levels of the TGF-beta receptor are increased in atherosclerosis. Atherosclerosis.

[8] Morikawa M., Derynck R., Miyazono K. (2016). TGF-β and the TGF-β Family: Context-Dependent Roles in Cell and Tissue Physiology. Cold Spring Harb. Perspect. Biol..

[9] Paradowska-Gorycka A., Roszak M., Stypinska B., Lutkowska A., Walczyk M., Olesinska M., Wajda A., Piotrowski P., Puszczewicz M., Majewski D. (2019). IL-6 and TGF-β gene polymorphisms, their serum levels, as well as HLA profile, in patients with systemic lupus erythematosus. Clin. Exp. Rheumatol..

[10] Stadtlober N.P., Flauzino T., Santos L.F.D.R.F., Iriyoda T.M.V., Costa N.T., Lozovoy M.A.B., Reiche E.M.V., Simão A.N.C. (2021). TGFB1 +869 T > C (rs1800470) variant is independently associated with susceptibility, laboratory activity, and TGF-β1 in patients with systemic lupus erythematosus. Autoimmunity.

[11] Shah R., Hurley C.K., Posch P.E. (2006). A molecular mechanism for the differential regulation of TGF-β1 expression due to the common SNP −509C-T (c. −1347C > T). Hum. Genet..

[12] Ser Ö.S., Çetinkal G., Kiliçarslan O., Dalgıç Y., Batit S., Keskin K., Özkara G., Aslan E.I., Aydoğan H.Y., Yıldız A. (2021). The comparison of serum TGF-beta levels and associated polymorphisms in patients with coronary artery ectasia and normal coronary artery. Egypt Heart J..

[13] Du L., Gong T., Yao M., Dai H., Ren H.G., Wang H. (2019). Contribution of the polymorphism rs1800469 of transforming growth factor β in the development of myocardial infarction: Meta-analysis of 5460 cases and 8413 controls (MOOSE-compliant article). Medicine.

[14] Morris D.R., Moxon J.V., Biros E., Krishna S.M., Golledge J. (2012). Meta-analysis of the association between transforming growth factor-beta polymorphisms and complications of coronary heart disease. PLoS ONE.

[15] Hu H.H., Chen D.Q., Wang Y.N., Feng Y.L., Cao G., Vaziri N.D., Zhao Y.Y. (2018). New insights into TGF-β/Smad signaling in tissue fibrosis. Chem. Biol. Interact..

[16] Chen C.L., Liu I.H., Fliesler S.J., Han X., Huang S.S., Huang J.S. (2007). Cholesterol suppresses cellular TGF-beta responsiveness: Implications in atherogenesis. J. Cell Sci..

[17] Reifenberg K., Cheng F., Orning C., Crain J., Küpper I., Wiese E., Protschka M., Blessing M., Lackner K.J., Torzewski M. (2012). Overexpression of TGF-ß1 in macrophages reduces and stabilizes atherosclerotic plaques in ApoE-deficient mice. PLoS ONE.

[18] Kang I., Yoon D.W., Braun K.R., Wight T.N. (2014). Expression of versican V3 by arterial smooth muscle cells alters tumor growth factor β (TGFβ)-, epidermal growth factor (EGF)-, and nuclear factor κB (NFκB)-dependent signaling pathways, creating a microenvironment that resists monocyte adhesion. J. Biol. Chem..

[19] Zhu Y., Xian X., Wang Z., Bi Y., Chen Q., Han X., Tang D., Chen R. (2018). Research Progress on the Relationship between Atherosclerosis and Inflammation. Biomolecules.

[20] Groenen A.G., Halmos B., Tall A.R., Westerterp M. (2021). Cholesterol efflux pathways, inflammation, and atherosclerosis. Crit. Rev. Biochem. Mol. Biol..

[21] Lin J., Kakkar V., Lu X. (2016). Essential Roles of Toll-Like Receptors in Atherosclerosis. Curr. Med. Chem..

[22] Silvestre-Roig C., Braster Q., Ortega-Gomez A., Soehnlein O. (2020). Neutrophils as regulators of cardiovascular inflammation. Nat. Rev. Cardiol..

[23] Klopf J., Brostjan C., Eilenberg W. (2021). Neumayer Neutrophil Extracellular Traps and Their Implications in Cardiovascular and Inflammatory Disease. Int. J. Mol. Sci..

[24] Döring Y., Soehnlein O., Weber C. (2017). Neutrophil Extracellular Traps in Atherosclerosis and Atherothrombosis. Circ. Res..

[25] Moschonas I.C., Tselepis A.D. (2019). The pathway of neutrophil extracellular traps towards atherosclerosis and thrombosis. Atherosclerosis.

[26] Nappi F., Bellomo F., Avtaar Singh S.S. (2022). Insights into the Role of Neutrophils and Neutrophil Extracellular Traps in Causing Cardiovascular Complications in Patients with COVID-19: A Systematic Review. J. Clin. Med..

[27] Koch W., Hoppmann P., Mueller J.C., Schömig A., Kastrati A. (2006). Association of transforming growth factor-beta1 gene polymorphisms with myocardial infarction in patients with angiographically proven coronary heart disease. Arterioscler. Thromb. Vasc. Biol..

[28] Chen Y., Dawes P.T., Packham J.C., Mattey D.L. (2012). Interaction between smoking and functional polymorphism in the TGFB1 gene is associated with ischaemic heart disease and myocardial infarction in patients with rheumatoid arthritis: A cross-sectional study. Arthritis Res. Ther..

[29] Xu J., Yu X., Huang C., Qin R., Peng F., Lin J., Niu W. (2015). Association of 5 Well-Defined Polymorphisms in the Gene Encoding Transforming Growth Factor-β1 with Coronary Artery Disease among Chinese Patients with Hypertension. Angiology.

[30] Yang M., Zhu M., Tang L., Zhu H., Lu Y., Xu B., Jiang J., Chen X. (2016). Polymorphisms of TGFβ-1 and TGFBR2 in relation to coronary artery disease in a Chinese population. Clin. Biochem..

[31] Duran J., Olavarría P.S., Mola M., Götzens V., Carballo J., Pelegrina E.M., Petit M., Abdul-Jawad O., Otaegui I., del Blanco B.G. (2015). Genetic association study of coronary collateral circulation in patients with coronary artery disease using 22 single nucleotide polymorphisms corresponding to 10 genes involved in postischemic neovascularization. BMC Cardiovasc. Disord..

[32] Lu Y., Boer J.M., Barsova R.M., Favorova O., Goel A., Müller M., Feskens E.J., PROCARDIS CARDIoGRAM Consortium (2012). TGFB1 genetic polymorphisms and coronary heart disease risk: A meta-analysis. BMC Med. Genet..

[33] Osadnik T., Strzelczyk J.K., Reguła R., Bujak K., Fronczek M., Gonera M., Gawlita M., Wasilewski J., Lekston A., Kurek A. (2016). The Relationships between Polymorphisms in Genes Encoding the Growth Factors TGF-β1, PDGFB, EGF, bFGF and VEGF-A and the Restenosis Process in Patients with Stable Coronary Artery Disease Treated with Bare Metal Stent. PLoS ONE.

[34] Fragoso J.M., Zuñiga-Ramos J., Arellano-González M., Alvarez-León E., Villegas-Torres B.E., Cruz-Lagunas A., Delgadillo-Rodriguez H., Peña-Duque M.A., Martínez-Ríos M.A., Vargas-Alarcón G. (2015). The T29C (rs1800470) polymorphism of the transforming growth factor-β1 (TGF-β1) gene is associated with restenosis after coronary stenting in Mexican patients. Exp. Mol. Pathol..

[35] Huang F., Li L., Shen C., Wang H., Chen J., Chen W., Chen X. (2014). Association between TGFBR2 gene polymorphisms and congenital heart defects in Han Chinese population. Nutr. Hosp..

[36] Tseng Z.H., Vittinghoff E., Musone S.L., Lin F., Whiteman D., Pawlikowska L., Kwok P.Y., Olgin J.E., Aouizerat B.E. (2009). Association of TGFBR2 polymorphism with risk of sudden cardiac arrest in patients with coronary artery disease. Heart Rhythm.

[37] Yakymovych I., Yakymovych M., Heldin C.H. (2018). Intracellular trafficking of transforming growth factor β receptors. Acta Biochim. Biophys. Sin..

[38] Iwata J., Suzuki A., Pelikan R.C., Ho T.V., Sanchez-Lara P.A., Chai Y. (2014). Modulation of lipid metabolic defects rescues cleft palate in Tgfbr2 mutant mice. Hum. Mol. Genet..

