# Supplementary material for: TGF-β1 and TGFβR2 Gene Polymorphisms in Patients with Unstable Angina
Source: Biomedicines. 2023 Jan 7;11(1):155. doi: 10.3390/biomedicines11010155 (PMC9855791; doi:10.3390/biomedicines11010155)
Supplement: Supplementary file 1 [file biomedicines-11-00155-s001.zip › biomedicines-2130558-supplementary.pdf]

Supplementary Tables

Supplementary Table S1. Distribution of *TGFB1* rs1800469, rs1800470, *TGFBR2* rs6785358, rs9838682 genotypes and alleles in with unstable angina and controls in < 55 years group.

	Control group (n = 15)		Unstable angina (n = 55)		p value^	Compared genotypes or alleles	p value#	OR (95% CI)
	n	%	n	%				
TGFB1 rs1800469								
**genotype**								
GG	7	46.67%	15	27.27%		AA+GA vs GG	0.21	2.33 (0.72–7.56)
GA	6	40.00%	35	63.64%	0.254	AA vs GA+GG	0.64	0.65 (0.11–3.74)
AA	2	13.33%	5	9.09%		AA vs GG	1.00	1.17 (0.18–7.56)
						GA vs GG	0.19	2.72 (0.78–9.47)
						AA vs GA	0.33	0.43 (0.07–2.74)
**Allele**								
G	20	66.67%	65	59.09%				
A	10	33.33%	45	40.91%		A vs G	0.53	1.39 (0.59–3.24)
TGFB1 rs1800470								
**genotype**								
GG	2	13.33%	9	16.36%		AA+GA vs GG	1.00	0.79 (0.15–4.10)
GA	7	46.67%	33	60.00%	0.450	AA vs GA+GG	0.33	0.46 (0.14–1.55)
AA	6	40.00%	13	23.64%		AA vs GG	0.67	0.48 (0.08–2.95)
						GA vs GG	1.00	1.05 (0.19–5.94)
						AA vs GA	0.31	0.46 (0.13–1.63)
**Allele**								
G	11	36.67%	51	46.36%		A vs G	0.41	0.67 (0.29–1.54)
A	19	63.33%	59	53.64%				
***TGFBR2* rs6785358**								
**genotype**								
AA	11	73.33%	41	74.54%		GG+AG vs AA	1.00	0.94 (0.26–3.43)
AG	3	20.00%	13	23.64%	0.594	GG vs AG+AA	0.39	0.26 (0.02–4.41)
GG	1	6.67%	1	1.82%		GG vs AA	0.40	0.27 (0.02–4.64)
						AG vs AA	1.00	1.16 (0.28–4.81)
						GG vs AG	0.41	0.23 (0.01–4.84)
**Allele**								
A	25	83.33%	95	86.36%		G vs A	0.77	0.79 (0.26–2.38)
G	5	16.67%	15	13.64%				
***TGFBR2* rs9838682**								
**genotype**								
AA	2	13.33%	4	7.27%		AA+AG vs GG	0.39	1.71 (0.54–5.41)
AG	5	33.33%	29	52.73%	0.386	AA vs AG+GG	0.60	0.51 (0.08–3.10)
GG	8	53.34%	22	40.00%		AA vs GG	1.00	0.73 (0.11–4.77)
						AG vs GG	0.35	2.11 (0.61–7.34)
**Allele**						AA vs AG	0.28	0.35 (0.05–2.41)
A	9	30.00%	37	33.64%				

G	21	70.00%	73	66.36%		A vs G	0.83	1.18 (0.49–2.84)

^ χ^2 test. # Fisher's Exact Test.

Supplementary Table S2. Distribution of *TGFB1* rs1800469, rs1800470, *TGFBR2* rs6785358, rs9838682 genotypes and alleles in with unstable angina and controls in ≥ 55 years group.

	Control group (*n* = 129)		Unstable angina (*n* = 177)		*p* value^	Compared genotypes or alleles	*p* value#	OR (95% CI)
	n	%	*n*	%				
TGFB1 rs1800469 **genotype**								
GG	55	42.64%	71	40.11%		AA+GA vs GG	0.72	1.11 (0.70–1.76)
GA	58	44.96%	83	46.89%	0.907	AA vs GA+GG	1.00	1.06 (0.53–2.09)
AA	16	12.40%	23	13.00%		AA vs GG	0.85	1.11 (0.54–2.31)
						GA vs GG	0.71	1.11 (0.68–1.80)
						AA vs GA	1.00	1.01 (0.49–2.07)
**Allele**								
G	168	65.12%	225	63.56%				
A	90	34.88%	129	36.44%		A vs G	0.73	1.07 (0.77–1.50)
TGFB1 rs1800470 **genotype**								
GG	22	17.05%	39	22.03%		GG+GA vs AA	0.90	0.96 (0.60–1.55)
GA	63	48.84%	76	42.94%	0.468	GG vs GA+AA	0.31	1.38 (0.77–2.46)
AA	44	34.11%	62	35.03%		GG vs AA	0.52	1.26 (0.66–2.41)
						GA vs AA	0.60	0.86 (0.51–1.43)
						GG vs GA	0.28	1.47 (0.79–2.73)
**Allele**								
G	107	41.47%	154	43.50%		G vs A	0.62	1.09 (0.79–1.50)
A	151	58.53%	200	56.50%				
TGFBR2 **rs6785358 genotype**								
AA	86	66.67%	132	74.58%		GG+AG vs AA	0.16	0.68 (0.41–1.12)
AG	42	32.56%	40	22.60%	0.081	GG vs AG+AA	0.41	3.72 (0.43–32.24)
GG	1	0.77%	5	2.82%		GG vs AA	0.41	3.26 (0.37–28.37)
						AG vs AA	0.07	0.62 (0.37–1.03)
						GG vs AG	0.20	5.25 (0.59–46.92)
**Allele**								
A	214	82.95%	304	85.88%		G vs A	0.36	0.80 (0.51–1.24)
G	44	17.05%	50	14.12%				
TGFBR2 **rs9838682 genotype**								
AA	18	13.95%	20	11.30%		AA+AG vs GG	0.20	0.72 (0.45–1.14)
AG	63	48.84%	77	43.50%	0.363	AA vs AG+GG	0.49	0.79 (0.40–1.55)
GG	48	37.21%	80	45.20%		AA vs GG	0.35	0.67 (0.32–1.38)
						AG vs GG	0.22	0.73 (0.45–1.20)
**Allele**						AA vs AG	0.86	0.91 (0.44–1.87)
A	99	38.37%	117	33.05%				
G	159	61.63%	237	66.95%		A vs G	0.20	0.79 (0.57–1.11)

^ χ^2 test. # Fisher's Exact Test.

Supplementary Table S3. Distributions of the *TGFB1* rs1800469, rs1800470, *TGFBR2* rs6785358, rs9838682 genotypes and alleles in unstable angina patients with and without diabetes mellitus (DM).

	Without Diabetes Mellitus (*n* = 175)		Diabetes Mellitus (*n* = 57)		*p*-value ^	Compared genotypes or alleles	*p*-value *	OR (95% CI)
	n	%	*n*	%				
TGFB1* *rs1800469								
**genotype**								
GG	65	37.14%	21	36.84%		AA+GA vs GG	1.00	1.01 (0.55–1.88)
GA	89	50.86%	29	50.88%	1.000	AA vs GA+GG	1.00	1.03 (0.41–2.56)
AA	21	12.00%	7	12.28%		AA vs GG	1.00	1.03 (0.39–2.77)
						GA vs GG	1.00	1.00 (0.53–1.93)
						AA vs GA	1.00	1.02 (0.40–2.65)
**Allele**								
G	219	62.57%	71	62.28%		A vs G	1.00	1.01 (0.66–1.57)
A	131	37.43%	43	37.72%				
TGFB1* *rs1800470								
**genotype**								
GG	39	22.29%	9	15.79%		GG+GA vs AA	1.00	1.05 (0.55–1.99)
GA	79	45.14%	30	52.63%	0.538	GG vs GA+AA	0.35	0.65 (0.30–1.45)
AA	57	32.57%	18	31.58%		GG vs AA	0.66	0.73 (0.30–1.79)
						GA vs AA	0.61	1.20 (0.61–2.37)
						GG vs GA	0.32	0.61 (0.26–1.41)
**Allele**								
G	157	44.86%	48	42.11%		G vs A	0.66	0.89 (0.58–1.37)
A	193	55.14%	66	57.89%				
***TGFBR2* rs6785358**								
**genotype**								
AA	130	74.29%	43	75.44%		GG+AG vs AA	1.00	0.94 (0.47–1.88)
AG	39	22.28%	14	24.56%	0.470	GG vs AG+AA	0.34	-
GG	6	3.43%	0	0.00%		GG vs AA	0.34	-
						AG vs AA	0.86	1.09 (0.54–2.19)
						GG vs AG	0.32	-
**Allele**								
A	299	85.43%	100	87.72%		G vs A	0.64	0.82 (0.44–1.55)
G	51	14.57%	14	12.28%				
***TGFBR2* rs9838682**								
**genotype**								
AA	18	10.29%	6	10.52%		AA+AG vs GG	0.76	1.11 (0.60–2.02)
AG	79	45.14%	27	47.37%	0.951	AA vs AG+GG	1.00	1.03 (0.39–2.73)
GG	78	44.57%	24	42.11%		AA vs GG	1.00	1.08 (0.39–3.04)
						AG vs GG	0.75	1.11 (0.59–2.09)
						AA vs AG	1.00	0.98 (0.35–2.71)
**Allele**								
A	115	32.86%	39	34.21%		A vs G	0.82	1.06 (0.68–1.66)
G	235	67.14%	75	65.79%				

^ χ2 test. *Fisher's exact test

Supplementary Table S4. Distributions of the *TGFB1* rs1800469, rs1800470, *TGFBR2* rs6785358, rs9838682 genotypes and alleles in unstable angina patients with and without arterial hypertension (HA).

	**Without Arterial Hypertension (*n* = 87)**		**Arterial Hypertension (*n* = 145)**		***p*-value ^**	**Compared genotypes or alleles**	***p*-value ***	**OR (95% CI)**
	n	**%**	***n***	**%**				
TGFB1* *rs1800469 **genotype**								
GG	32	36.78%	54	37.24%		AA+GA vs GG	1.00	0.98 (0.57–1.70)
GA	44	50.58%	74	51.04%	0.981	AA vs GA+GG	0.84	0.92 (0.41–2.06)
AA	11	12.64%	17	11.72%		AA vs GG	1.00	0.91 (0.38–2.20)
						GA vs GG	1.00	1.00 (0.56–1.77)
						AA vs GA	0.83	0.92 (0.40–2.14)
**Allele**								
G	108	62.07%	182	62.76%		A vs G	0.92	0.97 (0.66–1.43)
A	66	37.93%	108	37.24%				
TGFB1* *rs1800470 **genotype**								
GG	15	17.24%	33	22.76%		GG+GA vs AA	0.89	1.08 (0.61–1.90)
GA	43	49.43%	66	45.52%	0.622	GG vs GA+AA	0.40	1.41 (0.72–2.79)
AA	29	33.33%	46	31.72%		GG vs AA	0.45	1.39 (0.64–2.99)
						GA vs AA	1.00	0.97 (0.53–1.77)
						GG vs GA	0.37	1.43 (0.70–2.95)
**Allele**								
G	73	41.95%	132	45.52%		G vs A	0.50	1.16 (0.79–1.69)
A	101	58.05%	158	54.48%				
***TGFBR2* rs6785358 genotype**								
AA	65	74.71%	108	74.48%		GG+AG vs AA	1.00	1.01 (0.55–1.87)
AG	21	24.14%	32	22.07%	0.630	GG vs AG+AA	0.41	3.07 (0.35–26.73)
GG	1	1.15%	5	3.45%		GG vs AA	0.42	3.01 (0.34–26.33)
						AG vs AA	0.87	0.92 (0.49–1.72)
						GG vs AG	0.40	3.28 (0.36–30.11)
**Allele**								
A	151	86.78%	248	85.52%		G vs A	0.78	1.11 (0.64–1.92)
G	23	13.22%	42	14.48%				
***TGFBR2* rs9838682 genotype**								
AA	6	6.90%	18	12.41%		AA+AG vs GG	0.79	0.91 (0.53–1.56)
AG	44	50.57%	62	42.76%	0.311	AA vs AG+GG	0.27	1.91 (0.73–5.02)
GG	37	42.53%	65	44.83%		AA vs GG	0.35	1.71 (0.62–4.68)
						AG vs GG	0.48	0.80 (0.46–1.40)
						AA vs AG	0.17	2.13 (0.78–5.80)
**Allele**								
A	56	32.18%	98	33.79%		A vs G	0.76	1.08 (0.72–1.61)

G	118	67.82%	192	66.21%

^ $\chi2$ test. *Fisher's exact test.
